# Role of Imaging Spectrum Along With Other Diagnostic Modalities in Rhino-Orbital-Cerebral Mucormycosis (ROCM)

**DOI:** 10.7759/cureus.53962

**Published:** 2024-02-10

**Authors:** Virendrakumar Meena, Sitaram Barath, Shikhar Singh, Prateek Jakhar, Tarang Patel

**Affiliations:** 1 Radiology, Geetanjali Medical College & Hospital, Udaipur, IND; 2 Radiology, Aarthi Scans and Lab, Delhi, IND; 3 Radiodiagnosis, Geetanjali Medical College & Hospital, Udaipur, IND; 4 Pathology, All India Institute of Medical Sciences (AIIMS) Rajkot, Rajkot, IND

**Keywords:** orbital involvement, intracranial spread, thick fungal hyphae, contrast enhanced mri, covid-19, black turbinate sign, rhino-orbito-cerebral mucormycosis

## Abstract

Objectives: Rhino-orbito-cerebral mucormycosis (ROCM), a rare angio-invasive fungal infection, had become a major outbreak during the second wave of the coronavirus disease (COVID-19) pandemic in India, with over 28,000 reported cases. The purpose of this study was to describe the imaging spectrum of ROCM, which may prove useful in prompt diagnosis, considering its grave prognosis in populations with a high load of immunosuppressed patients (e.g., COVID-19, HIV-AIDS, etc.).

Material and methods: Evaluation of the clinical data and imaging of patients with symptoms suspicious of mucormycosis of the craniofacial region was done. The diagnosis was made using computed tomography (CT) or magnetic resonance (MR) imaging, a biopsy, and culture. The data analysis was done using descriptive statistical methods.

Results: The sample group consisted of a total of 36 patients ranging from 33 years to 75 years of age, out of which 31 (86.11%) were male and five (13.8%) were female. A total of 30 (83.33%) patients had a positive correlation with COVID-19 infection, and 29 (80.55%) patients had a positive correlation with diabetes. The major presenting complaints were facial pain and swelling (20 patients; 55.55%). The intracranial spread was seen in 14 (38.88%) patients. Our study demonstrated a mortality rate of 38.88% (14 patients).

Conclusion: ROCM, once considered to occur predominantly in diabetics, is increasingly being seen in other immunosuppressive patients, such as COVID-19. CT and MR imaging help provide an early diagnosis in conjunction with pathologic and microbiological correlations. Immediate correction of immunosuppression with the initiation of amphotericin B therapy combined with extensive and diligent surgical debridement of the diseased tissue is required.

## Introduction

Mucormycosis is an infection caused by fungi of the order Mucorales of the class Zygomycetes and was first described in 1885 by Paulltauf [1]. It became a major outbreak during the second wave of the coronavirus disease (COVID-19) pandemic in India, with over 28,000 reported cases [2,3]. They are a vasotropic species, resulting in tissue ischemia. The mucormycosis spectrum can involve the skin, paranasal sinus, cranium, orbit, and lungs, or a combination of them. It can cause widely disseminated and often deadly infections in individuals with low immunity [4].

The predominant sites of primary infection by invasive mucormycosis are the paranasal sinuses (39%), lungs (24%), and skin (19%). [5]. Diabetes mellitus is associated with mucormycosis cases to the tune of 36%-88% [6-8]. Patients with poorly controlled diabetes are at a higher risk [9]. The fungal sporangiospores, upon inhalation, enter the paranasal sinuses, followed by germination. The infection can rapidly spread to the palate, the sphenoid sinus, the cavernous sinus, the orbits, or the brain [10]. Intracranial spread pathways are through the orbital apex, or cribriform plate, of the ethmoid bone. Occasionally, it can involve the cerebral vessels, leading to the hematogenous spread of the infection, with the wall involvement causing infected pseudoaneurysms [11]. The initial symptoms of rhino-orbito-cerebral mucormycosis (ROCM) include sinusitis, inflammation around the eye, pain around the eye and/or facial pain, and decreased sensation on the face, followed by visual disturbances. Further signs and symptoms include multiple cranial nerve palsies, pain around the orbit, an inflamed orbit, eyelid edema, blepharoptosis, proptosis, restricted extraocular muscle movements, headaches, and loss of sight [12].

Imaging studies such as the computed tomography (CT) scan reveal the edematous mucosa, fluid-filled sinuses, and destruction of orbital soft tissues and bones. Although it is the imaging modality of choice for evaluating signs of infiltration, bone desolation happens late after soft-tissue necrosis has occurred [13]. Intracranial spread and vascular involvement are best studied using magnetic resonance (MR) imaging. Contrast-enhanced MR imaging better delineates the spread around nerves. MRI is more sensitive in detecting orbital soft tissue infection compared to CT scans [14]. Both CT scans and MR images can be normal in early cases; patients with a high risk for infection should be evaluated by surgical examination with a biopsy of the suspected areas of infection.

The aggressive nature and high fatality of intracranial involvement by ROCM warrant early radiological imaging followed by nasal endoscopy to rule out mucormycosis in diabetic patients presenting with headache and vision disturbances [4]. The main aim of the study was to narrate the role of CT and MRI findings in cases of ROCM, which can be utilized for the timely diagnosis of this disease in patients with compromised immunity.

## Materials and methods

Source of data

We evaluated the clinical data and imaging of patients presenting with mucormycosis of the craniofacial region at a tertiary care hospital in southern Rajasthan, India. Those patients whose diagnosis of mucormycosis was confirmed by biopsy, culture, and CT scans or MR images were selected for the study.

Study design

This is a retrospective observational study.

Study duration

The study was conducted from February 2021 to August 2021.

Methodology

We retrospectively analyzed clinical and imaging data of 36 biopsy-proven cases of rhino-orbital-cerebral mucormycosis from February 2021 to June 2021 after clearance from an institutional scientific and ethics review committee at a tertiary care hospital in southern Rajasthan, India. Microbiological or histological evidence of an invasive sinonasal fungal infection by a species from the order Mucorales was present in all the included individuals. Histopathological examination was conducted on hematoxylin- and eosin-stained paraffin-embedded sections.

Every patient had a history of COVID-19 disease, which was verified by a reverse transcriptase-polymerase chain reaction of a nasopharyngeal swab or high-resolution computed tomography of the chest. Patients who did not have complete radiological investigations were not included in our study. By looking through the patient's medical records, a thorough history of the patient and the results of the physical examination were gathered. All patients had their contrast computed tomography imaging on a 64-slice volume scanner (SIEMENS SOMATOM DEFINITION), and images were reformatted in the axial, coronal, and sagittal planes in soft tissue and in the bone window. MR imaging was done with a 3 Tesla scanner (SIGNA ARCHITECT). All patients had the MRI evaluations completed by the following sequences: unenhanced axial, sagittal, and coronal T1- and T2-weighted images and fat-saturated T1- and T2-weighted images. MR evaluation also included contrast-enhanced and diffusion-weighted imaging (DWI) with an apparent diffusion coefficient (ADC) map. The radiological findings were reviewed by radiologists. Tissue density, signal intensity, bone involvement, and contrast enhancement characteristics were assessed. On the T1WI and T2WI MRI signals, intensity was compared with gray matter for brain imaging and skeletal muscle for body imaging. Statistical analysis was done by using descriptive statistics. Means were used to provide a descriptive analysis of quantitative parameters. Ordinal data was expressed as an absolute number and a percentage.

Inclusion criteria

Those patients whose diagnosis of mucormycosis was confirmed by biopsy, culture, and CT scans or MR images were selected for the study.

Exclusion criteria

Patients who didn't have complete radiological investigations.

Assessment done

The radiological findings were reviewed by three radiologists. Tissue density, signal intensity, and contrast enhancement characteristics were assessed. On the T1WI and T2WI MRI signals, intensity was compared with gray matter for brain imaging and skeletal muscle for body imaging.

Statistical analysis

Statistical analysis was done by using descriptive statistics. Means were used to provide a descriptive analysis of quantitative parameters. Ordinal data was expressed as an absolute number and a percentage.

Ethical considerations

Ethical clearance was obtained from the institutional human research ethics committee.

## Results

A total of 36 patients suspected of ROCM were selected for this study. After undergoing a clinical examination and initial workup, the patients underwent MRI and CT imaging. The final confirmation of the diagnosis was done by histopathology.

Demographic and clinical findings

The sample group consisted of a total of 36 patients, with 31 (86.11%) male and five (13.8%) female patients. The patient was aged between 33 and 75 years, with a mean age of 49.5 years for the sample group.

As many as 30 (83.33%) patients had a COVID-19 infection or had a history of a recently treated COVID-19 infection, while six (16.66%) patients had no such history. The tally of patients who received systemic steroid therapy during COVID-19 treatment stands at 18 (50%). There were 14 patients with no history of steroid administration, and four patients were unable to provide the details.

Amongst the other predisposing conditions, a positive correlation with diabetes was present in 29 (80.55%) patients. Out of these 29 cases, 16 (44.44%) patients were known cases of diabetes, while 13 patients had recent-onset diabetes. Seven (19.44%) patients presented without diabetes. Out of the seven non-diabetic patients, two patients (5.55%) had accompanying malignant conditions (one with carcinoma rectum and another with carcinoma cervix), one (2.77%) patient was a known case of chronic myeloid leukemia, three (8.33%) patients had cirrhosis of the liver, and one (2.77%) patient had a history of pulmonary tuberculosis.

The major presenting complaints in our sample group were facial pain and swelling (20; 55.55%), proptosis (6; 16.66%), generalized weakness, fever and cough (6; 16.66% each), facial paralysis (3; 8.33%), eschar formation, difficulty chewing, multiple cranial palsies and eye pain (2; 5.5% each), and diplopia and decreased vision (1; 2.77% each).

Imaging findings

Sino-Nasal Involvement

**Figure 1:**
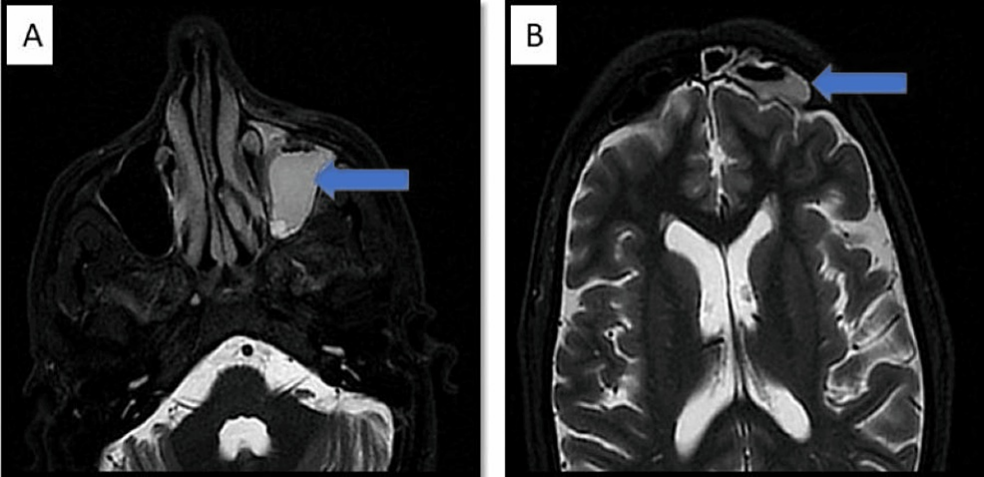
Sinonasal involvement Image A and B: T2 stir axial MR image shows mucosal thickening noted involving left maxillary, ethmoidal, and frontal sinuses, with these sinuses showing air fluid level.

In the cases included in our study, the most common pattern of involvement of the paranasal sinuses was the combination of maxillary, ethmoid, and sphenoid sinuses (13 patients; 36.11%). The bilateral involvement of paranasal sinuses was more common (22 patients; 61.11%). The disease extending to multiple paranasal sinuses seemed to be a common occurrence, with only one (2.77%) patient showing abnormal mucosal thickening in only the maxillary sinus. The seven (19.44%) patients had a pan-sinus involvement. The spectrum of sino-nasal disease included mild thickening of the mucosa, hypertrophied turbinates, inflammatory fluid accumulation, and bone erosion, as illustrated in Figure 1.

**Figure 2:**
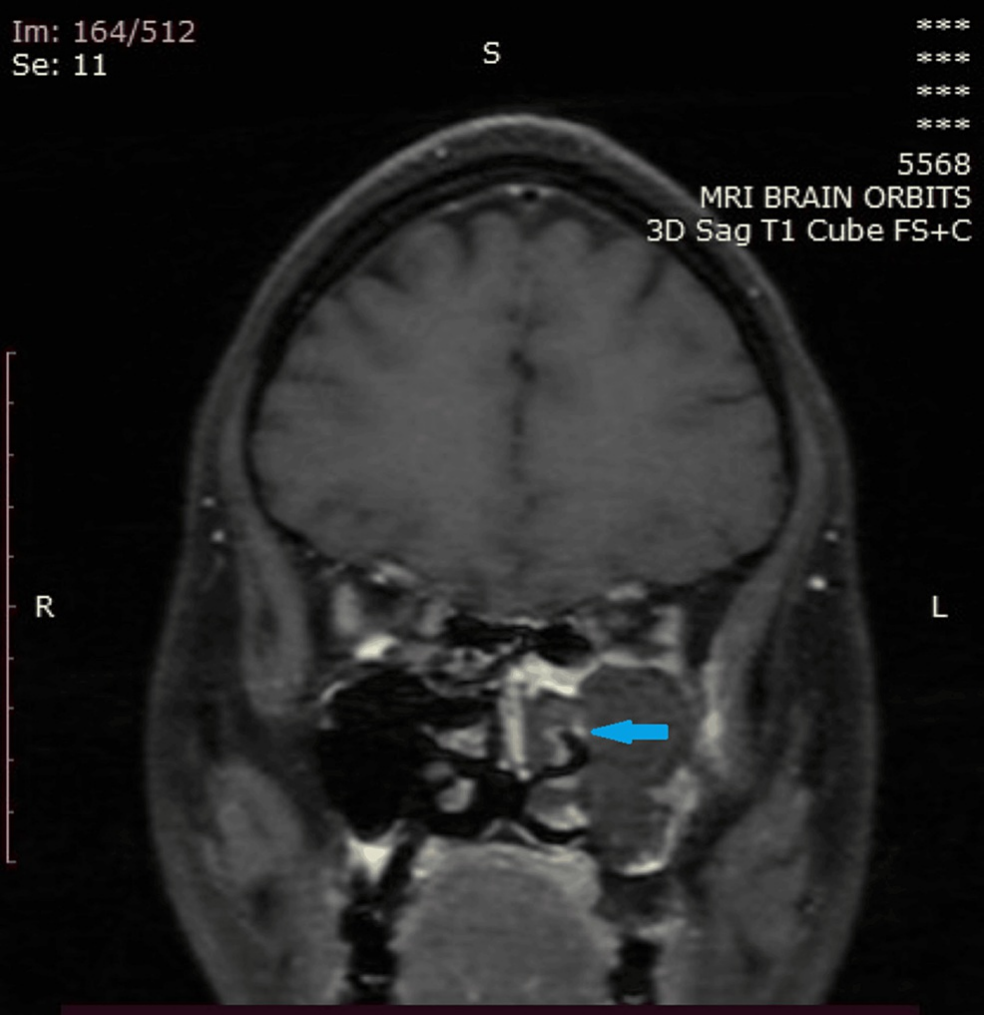
Sinonasal involvement Postcontrast T1-weighted fat-saturated coronal MR image shows non-enhancement of the middle and inferior turbinate left nasal cavity, showing a black turbinate sign and also being associated with left-sided maxillary sinusitis.

The "black turbinate sign" described by Safder et al. [15] was seen in five patients. The necrosis of tissue was identified as an absence of contrast enhancement in angioinvasive fungal sinusitis. The commonest location of black turbinate was the posterior aspect of the inferior turbinate [16]. These findings are demonstrated in Figure 2. 

Extra Sinus Involvement

In addition to the sino-nasal disease, we came across the involvement of the adjacent soft tissue as well as orbital and intracranial extension. The most common areas of involvement in extra sinus disease are the orbit (19 patients; 52.7%), infratemporal fossa (18 patients; 50%), and face (12 patients; 33.3%).

**Figure 3:**
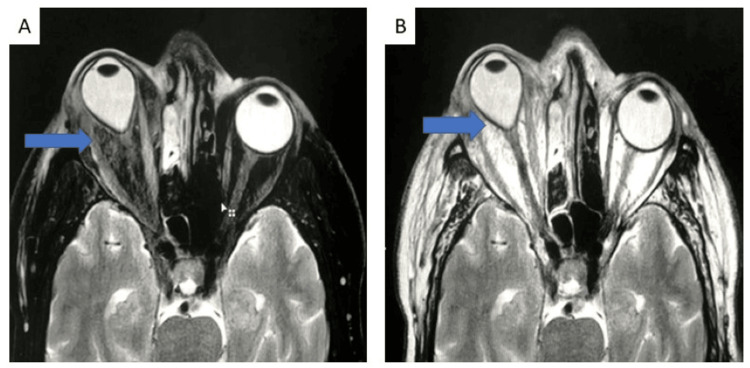
Orbital involvement: case 1 Axial MR image (T2FS/T2 sequences) shows thickening of lateral and medial rectus muscles with extensive fat stranding in the intra- and extraconal regions and muscle edema, resulting in distortion of the right globe (guitar pick sign) with proptosis.

**Figure 4:**
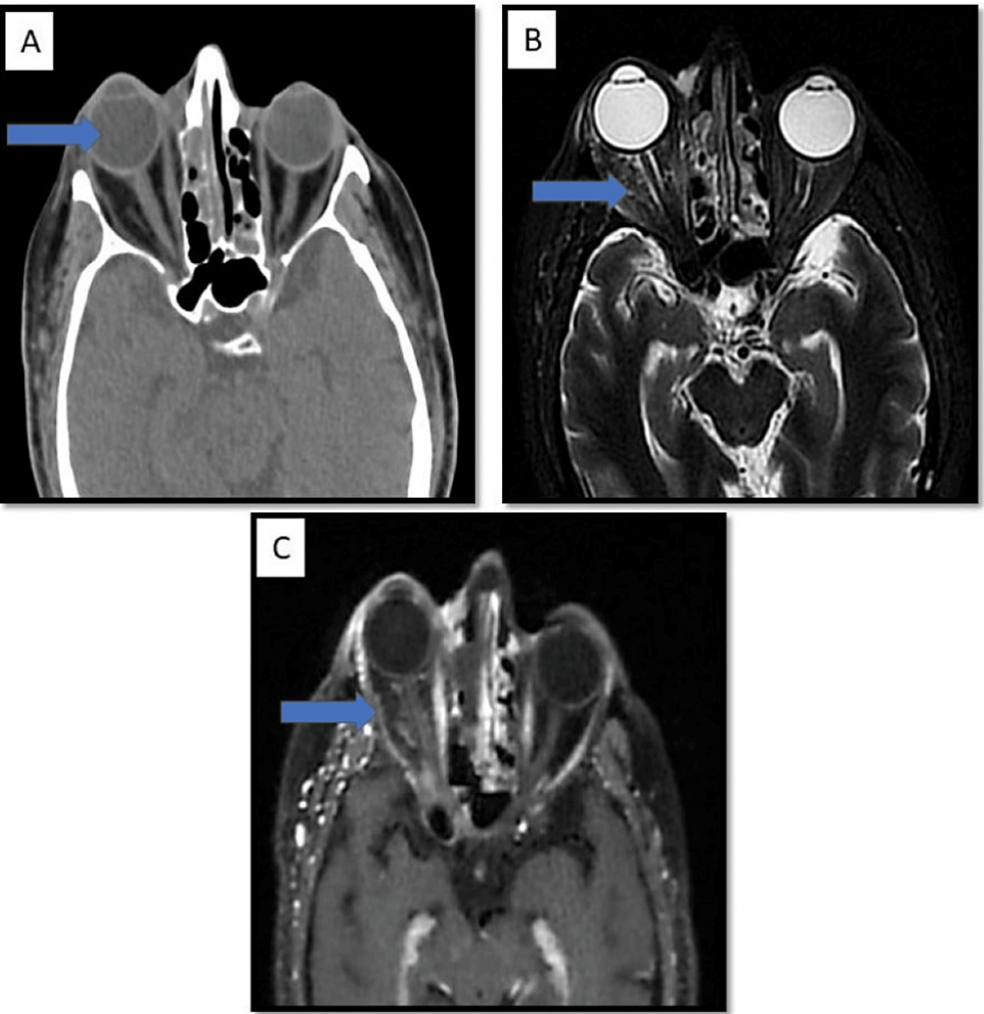
Orbital involvement: case 2 Image A: an axial CT image, shows proptosis of the right eye globe. Image B: T2 Stir-axial MR image shows fat stranding noted involving the intraconal orbital fat. Image C: Enhancement of medial rectus muscle and pre- and post-septal compartments of the right orbit on a post-contrast study

**Figure 5:**
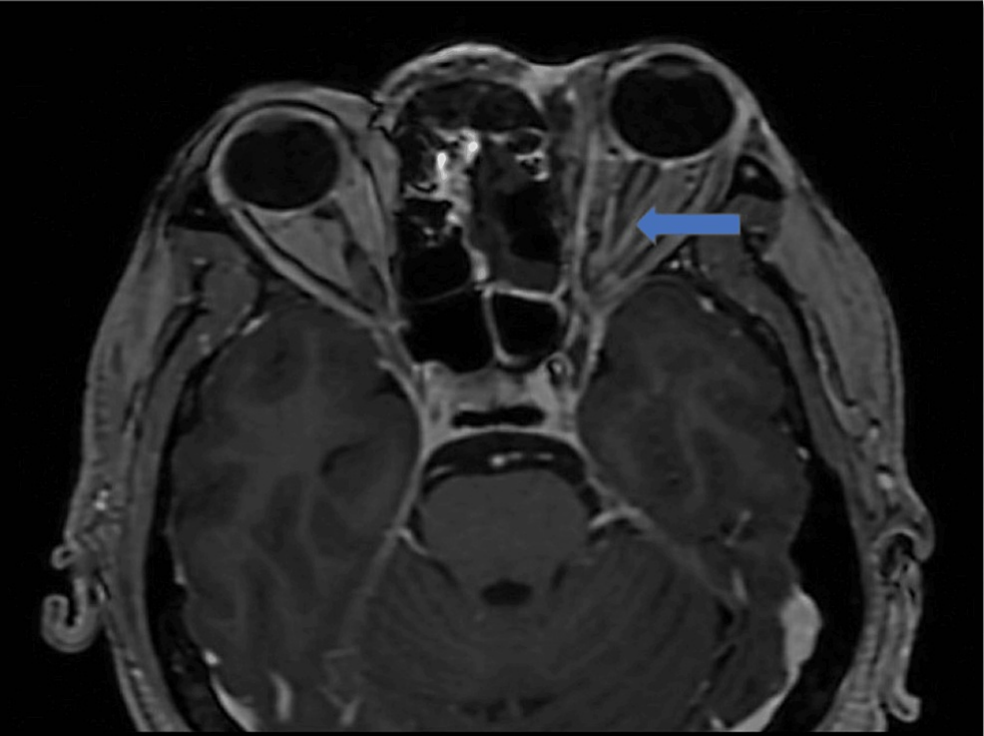
Orbital involvement: case 3 The axial T1 Bravo sequence shows significant enhancement in the left periorbital region and in the retro-orbital region, with significant patchy enhancement in the peri-optic nerve sheath and extending up to the orbital apex region.

Orbital involvement: The retrobulbar inflammation leads to excessive retrobulbar pressure and stretching of the optic nerve, causing the posterior globe to assume a 'guitar pick shape'. This condition is associated with acute and permanent visual damage and thus warrants prompt treatment (Figure 3: case 1), (Figure 4: case 2), and (Figure 5: case 3).

**Figure 6:**
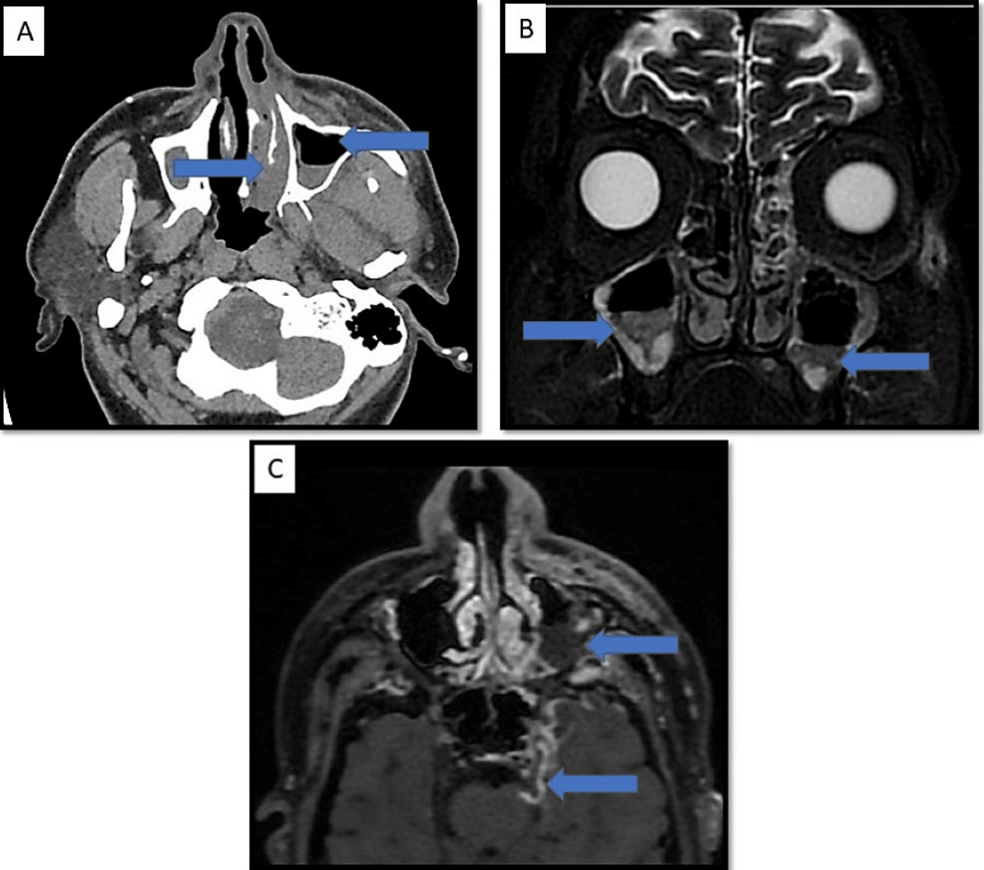
Intracranial involvement: case 1 A 33-year-old male of post-COVID status presented with left-sided facial pain and swelling. Image A-AXIAL CT image shows mucosal thickening involving the left middle turbinate and mild mucosal thickening with fluid level in the left maxillary sinus. The patient was classified as stage 2 ROCM and sent for a biopsy. The biopsy was negative for an invasive fungal infection. However, the same patient underwent MRI imaging 10 days later. Image B: Coronal T2 STIR image shows evidence of significant mucosal thickening and fluid collection in bilateral maxillary sinuses (L>R), left ethmoid air cells, and also in the left frontal sinus. Image C: Axial T1 post-contrast image shows involvement of the left Meckel’s cave and trigeminal nerve up to the pre-pontine cistern. These findings are consistent with perineural infiltration and cranial nerve involvement.

**Figure 7:**
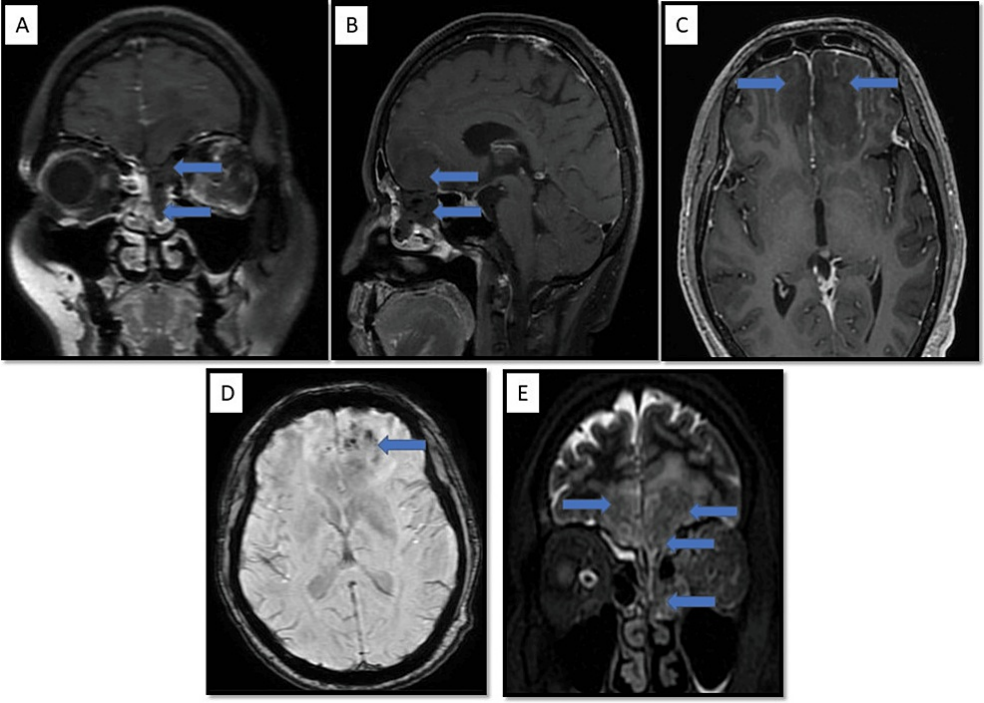
Intracranial involvement: case 2 There is evidence of bilateral ethmoidal (L>R) and left frontal sinusitis associated with significant extension and infiltration into the brain parenchyma in the bilateral basal-frontal region (L>R, which appears hypointense on the T2 sequence with multiple blooming foci in the left frontal region and shows peripheral diffusion restriction).

**Figure 8:**
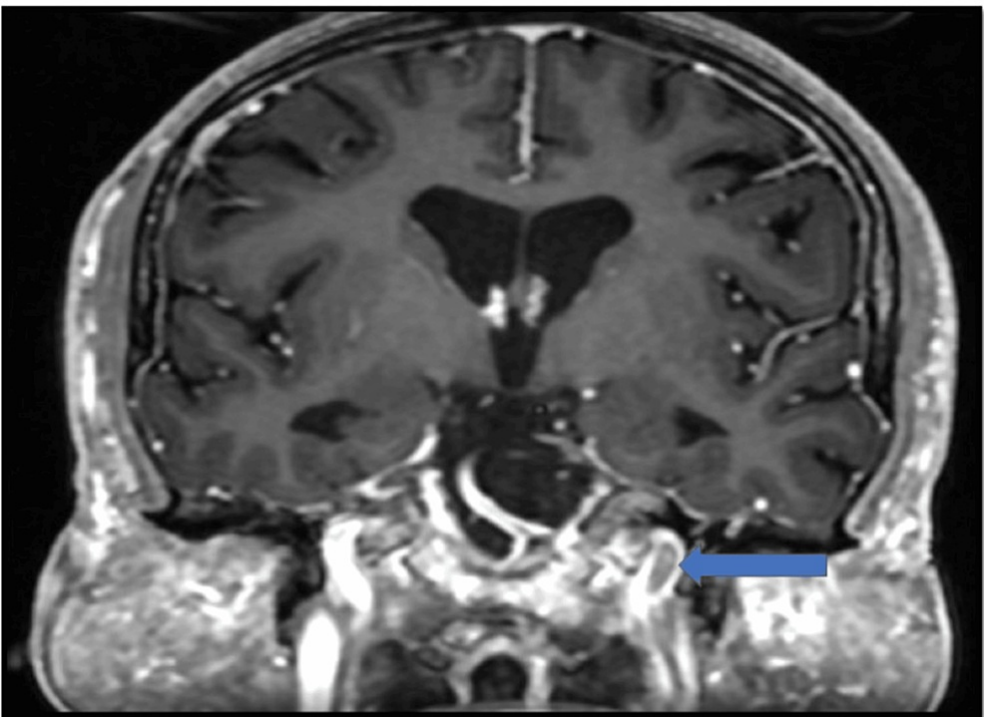
Intracranial involvement: case 3 This coronal post-contrast T1 image shows the absence of flow signal in the intracranial portion of the left internal carotid artery.

Intracranial involvement: Intracranial spread of disease was seen in 14 patients, with six patients showing involvement of brain parenchyma in the form of cerebritis, abscess, or infarcts, while thrombosis of the cavernous sinus was seen in four patients (Figure 6: Case 1), (Figure 7: Case 2), (Figure 8: Case 3).

**Figure 9:**
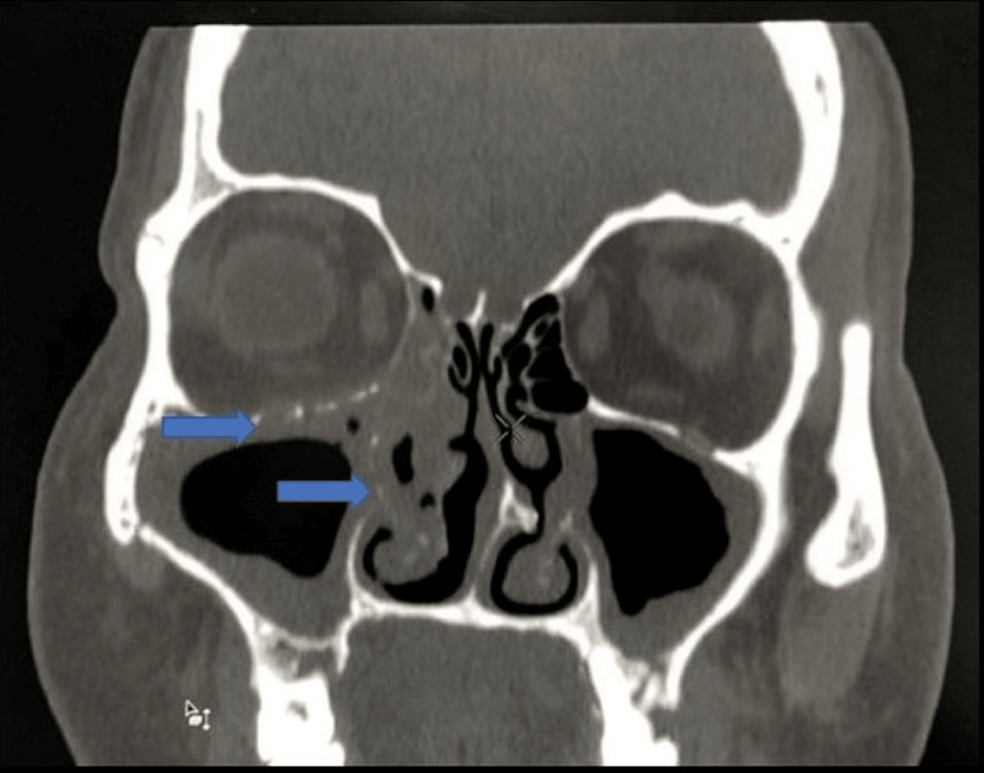
Bone involvement: case 1 The coronal section of this CT image shows mucosal thickening involving both maxillary sinuses, the right nasal cavity, and a blocked right osteomeatal complex with a permeative pattern in the inferior wall of the right orbit, the right lamina papyracea, the middle and inferior turbinate processes, and the right uncinate process, suggesting bony erosion. There is stranding in the right orbital fat involving both extraconal and intraconal compartments.

**Figure 10:**
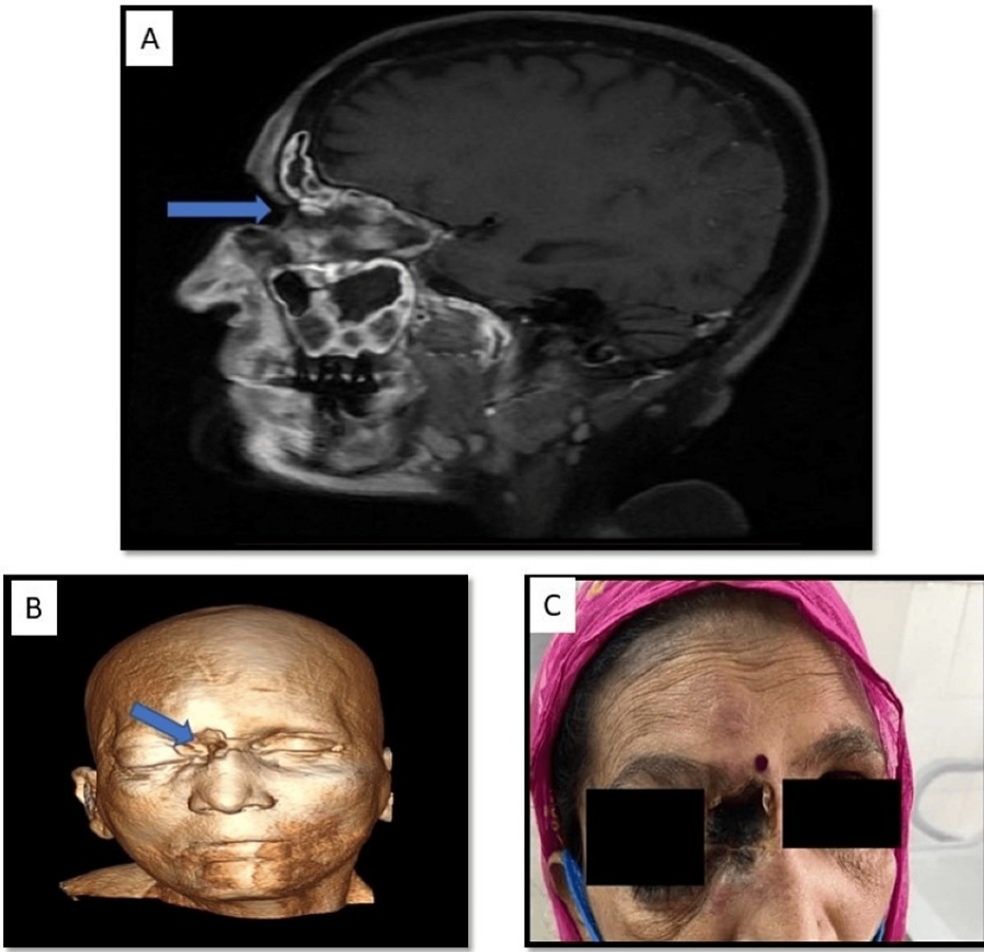
Bone involvement: case 2 Image A: Sagittal post-contrast T1 image shows soft tissue swelling, showing enhancement noted involving the subcutaneous plane of the right orbit, nose, and frontal region with a fistulous communication between the orbit and right paramedian frontal region opening to the external surface. Image B: 3D volume reconstruction. Image C: Gross image.

Bony involvement: Bone erosions and permeative desolation were seen in 10 cases (27.7%%) involving the bony wall of the sinuses (Figure 9: case 1), (Figure 10: case 2).

**Figure 11:**
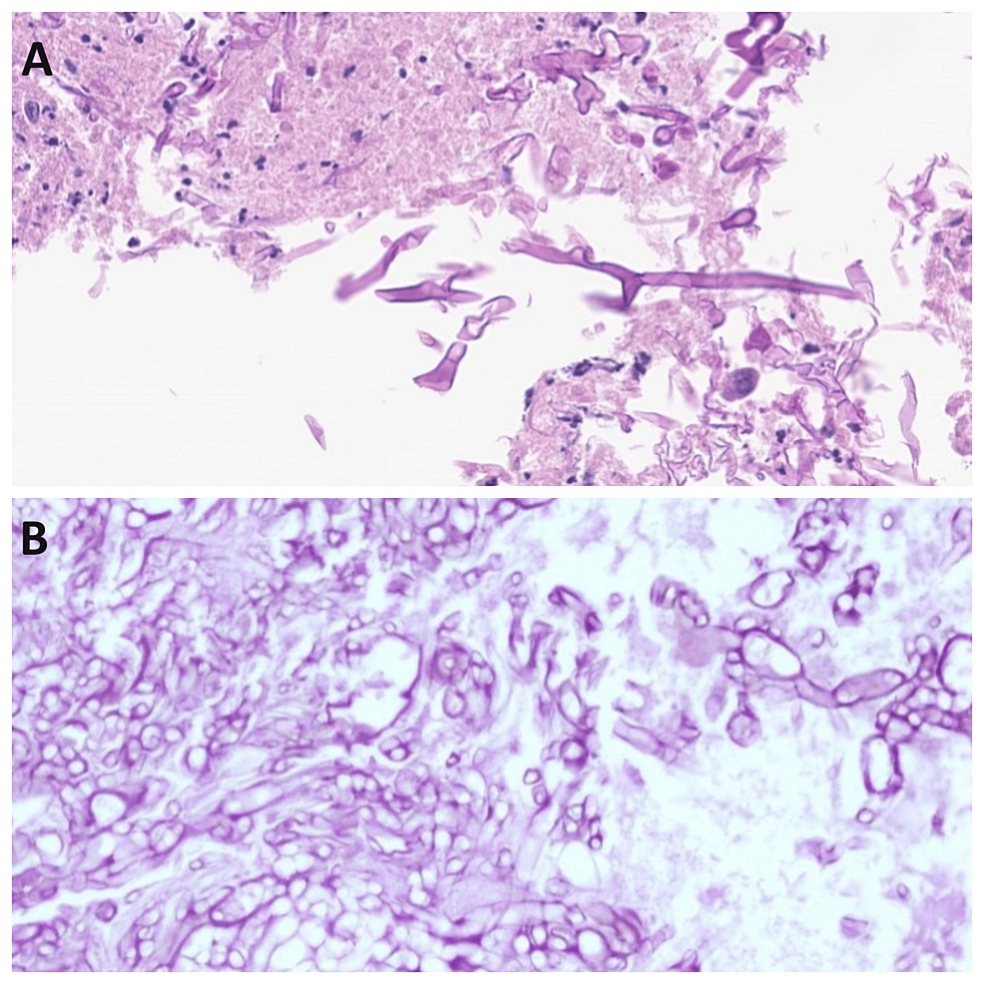
Histopathology Image A. Sinonasal and Image B. Orbital; mucormycosis showing thick aseptate hyphae with wide angel branching pattern under microscope (H&E stain, 40x)

According to the model for ROCM staging provided by Honavar SG et al. [17], in our study group, -14 patients (38.88%) were diagnosed at stage 2, eight patients (22.22%) at stage 3, and 14 patients (38.88%) at stage 4. Histopathology examination of the ROCM cases shows thick fungal hyphae with rare septations and obtuse angle branching. They were distinguished from aspergillosis on H&E stain, as later shows thin hyphae with septations and acute angle branching pattern (Figure 11).

The antifungal treatment (liposomal/conventional amphotericin B) was received by 29 patients (80.55%). Amongst our study group, 14 (38.88%) patients ultimately succumbed to rhino-orbito-cerebral mucormycosis, while 22 (61.11%) survived.

## Discussion

The term "Rhino-Orbito-Cerebral Mucormycosis (ROCM)" was first coined in 1955, and it emerged that the first target of mucor was the nasal mucosa. The infection can reach the brain via orbit or directly from the nasal cavity (i.e., rhinocerebral mucormycosis [RCM]). [18,19]. The incidence of intracranial mucormycosis was low, as reported by Talmi et al. [20]. However, during the second wave of the COVID-19 pandemic in India, mucormycosis emerged as a dangerous fungal infection with high mortality rates.

The order of "Mucorales" are ubiquitous fast-growing saprophytic aerobic microorganisms. The sporangiospores enter the nasal cavity and paranasal sinuses, where they invade the mucosa and undergo germination [15]. Upon entering the tissue, invasion of local arteries takes place, and the fungus adheres to the internal elastic lamina. This can result in thrombosis with progression to ischemia and necrosis of tissues (dry gangrene) [21]. The infection can travel beyond the boundaries of rhino-sinusal cavities with further involvement of the pterygopalatine fossa, periantral fat, nasolacrimal duct, the lacrimal sac, and, less commonly, the nasopharynx [22].

The subsequent orbital spread is orchestrated by the vascular embarrassment of orbital contents via the ethmoid arteries and the sphenopalatine artery. During this phase, the lymphatics and regional nerves can be infiltrated. Additional pathways of extension to orbit consist of spreading through the nasolacrimal duct and medial orbital wall [15,21]. Several pathways can lead to cerebral injury in "ROCM". The fungus can spread to the frontal lobe via the ophthalmic arteries and cribriform plate or to the cavernous sinus via the orbital apex. It can also be a manifestation of ischemia secondary to internal carotid artery occlusion due to "fibrin reaction and mucor thrombus formation" [15]. The perineural and perivascular spread have been described previously by Galletta K et al., with several cases from our study group demonstrating the same findings [22].

The regular activity of phagocytes is well known to help in fending off fungal infections by killing the spores. It is seen that reduced phagocytic activity and/or neutropenia are the two conditions that hastened opportunistic mold infections [23]. Several conditions are implicated in creating a predisposition to mucormycosis, such as malignant hematological disease, poorly controlled diabetes mellitus, major trauma, prolonged use of corticosteroids, intravenous drug abuse, and malnutrition [4]. Diabetes has a severely skewed worldwide distribution, with diabetes-related deaths in poor countries accounting for about 80%. Emerging countries such as India show higher numbers among urban populations than rural populations [24]. The largest literature review of mucormycosis from 1885 to 2004 by Roden MM et al. showed 36% (n = 337) of the 929 proven or probable cases diagnosed with DM [6]. Chakrabarti et al. showed uncontrolled diabetes as the commonest underlying cause in 74% (n = 131) of their 178 cases identified between 2000 and 2004 in India [25]. Our study shows a positive correlation in 29 (80.55%) patients. The Zygomycetes show increased survival in diabetic ketoacidosis patients due to their innate ketone reductase system. The diminished phagocytosis and more free iron in the blood of diabetic patients provide a more hospitable environment for the fungi [26].

The 2019 "novel coronavirus disease (COVID-19)" caused by the "severe acute respiratory syndrome coronavirus 2 (SARS-CoV-2)" is an infectious viral disease known to cause lower respiratory involvement, ranging from mild to severe pneumonia. It is also associated with bacterial and fungal co-infections. [27]. A high-dose steroid used in COVID-19 patients for a long duration increases the risk of developing mucormycosis by precipitating novel diabetes. In our study, 13 out of 36 patients had novel diabetes. More thrombus formation in these patients increased the iron availability of the fungus and, thus, their growth. An increased serum ferritin level combined with a decrease in the iron-binding capacity of transferrin in patients with ketoacidosis with COVID-19 infection can provide the iron needed for the growth of the fungi [28]. In our study, 30 (83.33%) patients with mucormycosis were diagnosed with COVID-19 infection or had a history of recently treated COVID-19 infection, compared to 5% in a study of 99 patients with COVID-19 infection by Chen et al. and 27.7% of patients by Michelle Bartoletti [28]. The use of antifungal agents not active against zygomycetes, for example, voriconazole and caspofungin, has also been thought to cause breakthrough zygomycosis [4].

Symptoms of patients presenting with mucormycosis include sinusitis with fever, nasal discharge, and headache. Surrounding structure involvement can occur very rapidly, i.e., orbit, palate, infratemporal region, and parapharyngeal region. A blackish eschar due to tissue necrosis occurs in the palate and nasal mucosa. Infection can also spread downward to the infratemporal fossa and parapharyngeal neck spaces, involving the 9th, 10th, 11th, and 12th cranial nerves. Orbital involvement presents with periorbital puffiness, redness, and orbital apex syndrome with the involvement of the 3rd, 4th, and 6th cranial nerves. Blindness is a possibility when an invasion of the optic canal occurs. The trigeminal nerve and facial nerve involvement occur by direct perineural spread as well as by vascular insult to the nerve [29].

Imaging features

An early diagnosis of ROCM can be made by identifying fungal invasion of craniofacial tissues on CT scans and MRIs. A preoperative contrast-enhanced CT scan may demonstrate the edematous mucosa, sinus opacification, air-fluid concentration, obliteration of the nasopharyngeal tissue planes, and destruction of periorbital tissues and bony margins. The fungal infection leads to the formation of heterogeneously enhanced anterior cranial fossa floor masses along with sinusoidal masses. MR Imaging findings consist of mucosal thickening along the paranasal sinuses, showing variable intensity on T1- and T2-weighted images. T2-weighted images may reveal the low signal intensity of fungal elements and restricted diffusion on DWI. The T2 hypointensity can be attributed to the iron and manganese present in the fungal elements. However, T2W signal or enhancement patterns can vary and are not reliable markers for invasive fungal infection. These patients can often exhibit subtle fat stranding in the premaxillary region, retromaxillary fat, orbital fat, and pterygopalatine fossa, which can be very important in making the diagnosis of an invasive fungal infection on imaging. Nasal secretions and nasal turbinate hypertrophy are seen with nasal involvement [15].

The thickened mucosa and involved tissues can show post-contrast enhancement. Contrast-enhanced MRI scans can also demonstrate the perineural spread of the disease [29]. Amongst the enhancing soft tissue of paranasal sinuses, there can be focal areas or widespread regions of non-enhancement. This is the diseased tissue prone to necrosis and devitalization and has been previously described by Safder et al. [15] as "the black turbinate” sign, which can aid in earlier detection of the disease. The blood vessel thrombosis caused by the fungus causing ischemia and necrosis of the brain shows diffusion restriction. Hence, DWI can differentiate other infiltrative lesions, such as nasopharyngeal carcinoma, from mucormycosis.

CT vs. MRI

CT imaging shows mucosal thickening and air-fluid levels in the early stages, with bony destruction becoming evident in later stages. MRI provides better visualization of the involvement of orbital soft tissue, infratemporal fossa, intracranial structures, perineural invasion, and vascular obstruction than CT due to better soft tissue resolution. Due to the patients receiving therapy with nephrotoxic drugs, compromised renal function can become worse from the potential nephrotoxicity of iodinated contrast use in CT [22]. However, in our protocol, we tried to utilize the advantages of both modalities by pairing a contrast-enhanced MRI with a non-contrast CT scan. A final diagnosis can be made by obtaining a biopsy of the involved tissue and being sent for histopathology as well as microbiological diagnostics. Necrotic tissue with non-septate hyphae, suppurative inflammation, and vascular thrombosis was described by Mohindra et al. [30].

Management

Early and extensive debridement to get clear margins, along with antifungal therapy, is important to stop the further spread of infection. Debridement at a later stage can be quite mutilating. Amphotericin B is the drug of choice for mucor infection. Liposomal amphotericin has four times faster serum level build-up and more tissue penetration and is less toxic as compared to conventional amphotericin therapy. The high cost remains a major limiting factor. The duration of therapy has not been determined but is generally continued until lesions resolve or stabilize. Posaconazole is given to patients who are intolerant of amphotericin B. Hyperbaric oxygen therapy helps in the oxidative metabolism of phagocytic cells and increases oxygenation of hypoxic tissue [29]. The death and disability rate of mucormycosis is high and can be fatal even within two weeks [15]. Patients undergoing debridement and amphotericin therapy have an overall better prognosis.

Limitations

Our study is also not without limitations. There is a lack of correlation studies between the clinical results and the imaging findings. The other limitation is the lack of randomization and blinding, which results in some confounding effects in the present study.

## Conclusions

ROCM, which was once considered to occur exclusively in diabetics, is increasingly being seen in other immunosuppressive patients, such as COVID-19. Diminished phagocytic activity with increased free iron availability in these patients due to diabetes, especially when associated with prolonged high-dose steroid usage, predisposes to an increased incidence of mucormycosis. When a pattern of nasal cavity, paranasal sinuses, and orbital inflammatory processes are present, CT and MR imaging help to provide an early diagnosis in conjunction with pathologic and microbiological correlation. Aggressive correction of immunosuppression and other predisposing factors with the initiation of amphotericin B therapy combined with extensive, diligent surgical debridement of the diseased tissues is required. However, despite radical surgery and an antifungal regimen, the prognosis is grave. Due to the emergence of numerous variants in the COVID-19 pandemic and the host of co-morbidities that come along, all radiologists should have a thorough knowledge of the imaging spectrum of "rhino-orbital mucormycosis" and its possible complications.

## References

[1] Paltauf A (1885). Mycosis mucorina. Archiv f. pathol. Anat..

[2] Adil A (2023). AA: Over 28,200 'black fungus' cases recorded In India. https://www.aa.com.tr/en/asia-pacific/over-28-200-black-fungus-cases-recorded-in-india/2266396.

[3] (2023). WHO: Weekly epidemiological update (3 November 2020). https://www.who.int/publications/m/item/weekly-epidemiological-update---3-november-2020.

[4] Petrikkos G, Skiada A, Lortholary O, Roilides E, Walsh TJ, Kontoyiannis DP (2012). Epidemiology and clinical manifestations of mucormycosis. Clin Infect Dis.

[5] Torres-Narbona M, Guinea J, Martínez-Alarcón J, Muñoz P, Gadea I, Bouza E (2007). Impact of zygomycosis on microbiology workload: a survey study in Spain. J Clin Microbiol.

[6] Roden MM, Zaoutis TE, Buchanan WL (2005). Epidemiology and outcome of zygomycosis: a review of 929 reported cases. Clin Infect Dis.

[7] Nithyanandam S, Jacob MS, Battu RR, Thomas RK, Correa MA, D'Souza O (2003). Rhino-orbito-cerebral mucormycosis. A retrospective analysis of clinical features and treatment outcomes. Indian J Ophthalmol.

[8] Ludvigsson J (2006). Why diabetes incidence increases--a unifying theory. Ann N Y Acad Sci.

[9] Gonzalez C, Rinaldi M, Sugar A (2002). Zygomycosis. Infect Dis Clin North Am.

[10] Hosseini SM, Borghei P (2005). Rhinocerebral mucormycosis: pathways of spread. Eur Arch Otorhinolaryngol.

[11] Orguc S, Yücetürk AV, Demir MA, Goktan C (2005). Rhinocerebral mucormycosis: perineural spread via the trigeminal nerve. J Clin Neurosci.

[12] Ribes JA, Vanover-Sams CL, Baker DJ (2000). Zygomycetes in human disease. Clin Microbiol Rev.

[13] Franquet T, Giménez A, Hidalgo A (2004). Imaging of opportunistic fungal infections in immunocompromised patient. Eur J Radiol.

[14] Garces P, Mueller D, Trevenen C (1994). Rhinocerebral mucormycosis in a child with leukemia: CT and MRI findings. Pediatr Radiol.

[15] Safder S, Carpenter JS, Roberts TD, Bailey N (2010). The "Black Turbinate" sign: An early MR imaging finding of nasal mucormycosis. AJNR Am J Neuroradiol.

[16] Han Q, Escott EJ (2019). The black turbinate sign, a potential diagnostic pitfall: evaluation of the normal enhancement patterns of the nasal turbinates. AJNR Am J Neuroradiol.

[17] Honavar SG (2021). Code mucor: guidelines for the diagnosis, staging and management of rhino-orbito-cerebral mucormycosis in the setting of COVID-19. Indian J Ophthalmol.

[18] Wali U, Balkhair A, Al-Mujaini A (2012). Cerebro-rhino orbital mucormycosis: an update. J Infect Public Health.

[19] Schell W (2000). Histopathology of fungal rhinosinusitis. Otolaryngol Clin North Am.

[20] Talmi YP, Goldschmied-Reouven A, Bakon M (2002). Rhino-orbital and rhino-orbito-cerebral mucormycosis. Otolaryngol Head Neck Surg.

[21] Pillsbury HC, Fischer ND (1977). Rhinocerebral mucormycosis. Arch Otolaryngol.

[22] Galletta K, Alafaci C, D'Alcontres FS (2021). Imaging features of perineural and perivascular spread in rapidly progressive rhino-orbital-cerebral mucormycosis: A case report and brief review of the literature. Surg Neurol Int.

[23] Bouza E, Muñoz P, Guinea J (2006). Mucormycosis: an emerging disease?. Clinical Microbiology and Infection.

[24] Diamond J (2011). Medicine: diabetes in India. Nature.

[25] Chakrabarti A, Das A, Mandal J (2006). The rising trend of invasive zygomycosis in patients with uncontrolled diabetes mellitus. Med Mycol.

[26] Rammaert B, Lanternier F, Poirée S, Kania R, Lortholary O (2012). Diabetes and mucormycosis: a complex interplay. Diabetes Metab.

[27] Awal SS, Biswas SS, Awal SK (2021). Rhino-orbital mucormycosis in COVID-19 patients—a new threat?.

[28] Eswaran S, Balan SK, Saravanam PK (2022). Acute fulminant Mucormycosis triggered by Covid 19 infection in a young patient. Indian J Otolaryngol Head Neck Surg.

[29] Narayanan S, Panarkandy G, Subramaniam G (2017). The "black evil" affecting patients with diabetes: a case of rhino orbito cerebral mucormycosis causing Garcin syndrome. Infect Drug Resist.

[30] Mohindra S, Mohindra S, Gupta R, Bakshi J, Gupta SK (2007). Rhinocerebral mucormycosis: the disease spectrum in 27 patients. Mycoses.

